# V-primer: software for the efficient design of genome-wide InDel and SNP markers from multi-sample variant call format (VCF) genotyping data

**DOI:** 10.1270/jsbbs.23018

**Published:** 2023-09-09

**Authors:** Satoshi Natsume, Kaori Oikawa, Chihiro Nomura, Kazue Ito, Hiroe Utsushi, Motoki Shimizu, Ryohei Terauchi, Akira Abe

**Affiliations:** 1 Department of Genomics and Breeding, Iwate Biotechnology Research Center, 22-174-4 Narita, Kitakami, Iwate 024-0003, Japan; 2 Crop Evolution Laboratory, Kyoto University, 1 Nakajo, Mozume, Muko, Kyoto 617-0001, Japan

**Keywords:** molecular marker, primer design, target amplicon sequencing, variant call format, next-generation sequencing, genomics, breeding

## Abstract

DNA markers are indispensable tools in genetics and genomics research as well as in crop breeding, particularly for marker-assisted selection. Recent advances in next-generation sequencing technology have made it easier to obtain genome sequences for various crop species, enabling the large-scale identification of DNA polymorphisms among varieties, which in turn has made DNA marker design more accessible. However, existing primer design software is not suitable for designing many types of genome-wide DNA markers from next-generation sequencing data. Here, we describe the development of V-primer, high-throughput software for designing insertion/deletion, cleaved amplified polymorphic sequence, and single-nucleotide polymorphism (SNP) markers. We validated the applicability of these markers in different crops. In addition, we performed multiplex PCR targeted amplicon sequencing using SNP markers designed with V-primer. Our results demonstrate that V-primer facilitates the efficient and accurate design of primers and is thus a useful tool for genetics, genomics, and crop breeding. V-primer is freely available at https://github.com/ncod3/vprimer.

## Introduction

DNA markers are indispensable tools in genetics and genomics research as well as in crop breeding, particularly in the context of marker-assisted selection. Insertion/deletion (InDel) and cleaved amplified polymorphic sequence (CAPS) markers are widely used genotyping tools due to their low cost and ease of use. These markers do not typically require expensive chemicals or equipment, making them widely accessible. Recent advances in next-generation sequencing (NGS) technology and the emergence of portal sites for plant genome information, such as “Plant GARDEN” (Kazusa DNA Research Institute, https://plantgarden.jp/en/index), have made it easier to obtain genome sequences for various crop species, enabling the large-scale identification of DNA polymorphisms among varieties, which, in turn, has made DNA marker design more accessible. However, existing software, such as Primer3 (Untergasser *et al.* 2012), can design primers for specific sequences but is not suitable for simultaneously designing large numbers of genome-wide DNA markers to detect polymorphisms among varieties.

High-throughput primer design programs have been recently developed to handle large-scale data output from NGS. For example, the mInDel pipeline (Lv *et al.* 2016) can design genome-wide InDel markers at high throughput by detecting InDels using *de novo* assembly of NGS short reads. However, this platform only supports InDel markers. Similarly, VCF2CAPS software (Wesołowski *et al.* 2021) can design CAPS markers at high throughput from variant call format (VCF) genotyping data derived from NGS, but only supports CAPS markers. No software comprehensively designs InDel and CAPS primers.

Reduced representation methods, which involve sequencing only a subset of the genome using NGS and collecting genotype information from those data, are effective techniques for handling many markers. Genotyping-by-sequencing technologies, such as restriction site–associated DNA sequencing (RADseq and ddRADseq) (Baird *et al.* 2008, Peterson *et al.* 2012) and genotyping by random amplicon sequencing-direct (GRAS-Di) (Enoki *et al.* 2018), sequence a small subset (less than 1%) of the genome, reducing the cost of genotyping per sample and allowing for more samples per run. However, a weakness of these methods is that they use random positions in the genome. By contrast, PCR-based reduced representation methods take advantage of the benefits of PCR and consistently amplify only single-nucleotide polymorphisms (SNPs) using specific PCR primers, resulting in high data reproducibility and robustness against SNPs. These PCR-based methods can use low-quality DNA and flexibly adjust the number of SNPs to be genotyped. Ion AmpliSeq technology (Thermo Fisher Scientific, Inc., USA), a PCR-based technique, is widely used in genetics research, including crop breeding, due to its flexibility in target region diversity and the number of SNPs and samples per analysis (Ogiso-Tanaka *et al.* 2019, Takeshima *et al.* 2021). In PCR-based target amplicon sequencing, primers used in the first multiplex PCR must be carefully designed to avoid secondary structures, dimer formation, and nonspecific amplification (Henegariu *et al.* 1997). However, few programs are available that can efficiently design hundreds of primers from the massive amount of data obtained by NGS (Yuan *et al.* 2021).

VCF (Danecek *et al.* 2011) files have become the standard format for representing genetic variation across individuals or populations using information obtained from NGS. These files are widely adopted as the input, intermediate output, and final output format for many tools used in genetic analysis using variant data. Here, we developed a high-throughput and efficient tool for designing InDel, CAPS, and SNP markers from VCF files of multi-sample variant data obtained by NGS. These markers can be used to perform DNA polymorphism analysis and multiplex PCR target amplicon sequencing and have applications in genetics, genomics, and breeding studies.

## Materials and Methods

### Design and implementation of V-primer

**Fig. 1.**
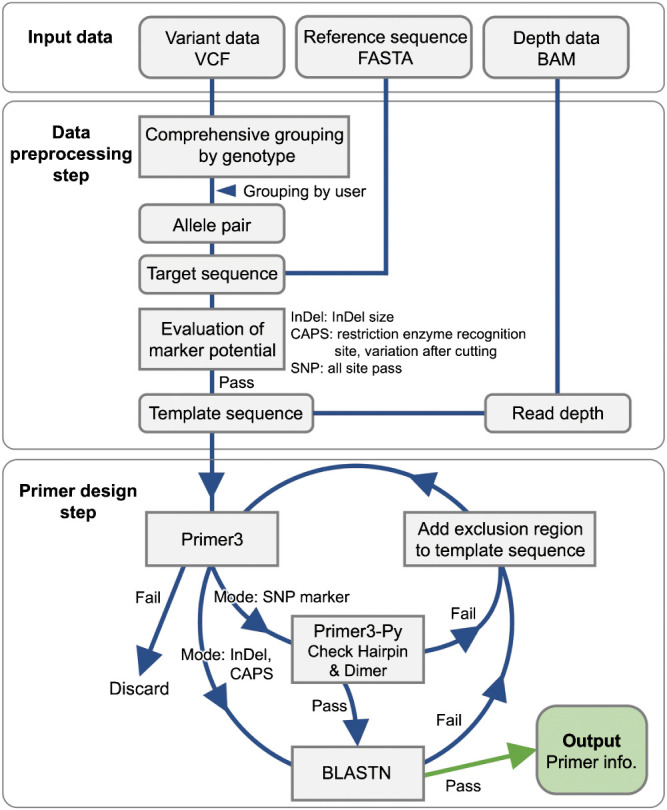
The V-primer workflow.

V-primer is a Python-based program executed through the command-line interface. The primer design algorithm of V-primer is based on Primer3 software (Untergasser *et al.* 2012) and requires other essential software tools like BLAST+ (Camacho *et al.* 2009), SAMtools (Li *et al.* 2009), BCFtools (Li 2011), BEDtools (https://bedtools.readthedocs.io/en/latest/), Python library VCFPy (https://vcfpy.readthedocs.io/en/stable/), Python library BioPython (Cock *et al.* 2009), and Python library Primer3-py (https://github.com/libnano/primer3-py). An overview of the workflow is presented in Fig. 1.

### Input files

V-primer requires two input files: a VCF file created by any variant calling software and a FASTA-formatted file containing the reference sequence to which the short reads were aligned during the generation of the VCF file. Any VCF format can be used if there is GT data in the FORMAT field. The VCF file can contain information on multiple samples, and users have the flexibility to select which samples to use for primer design, rather than having to use all the samples contained in the VCF file. Additionally, a BAM-formatted file generated during short-read alignment and VCF file creation can also be used as input. The read depth information from BAM files allows for more accurate primer design, so we recommend using a BAM file if possible.

### Data preprocessing

**Fig. 2.**
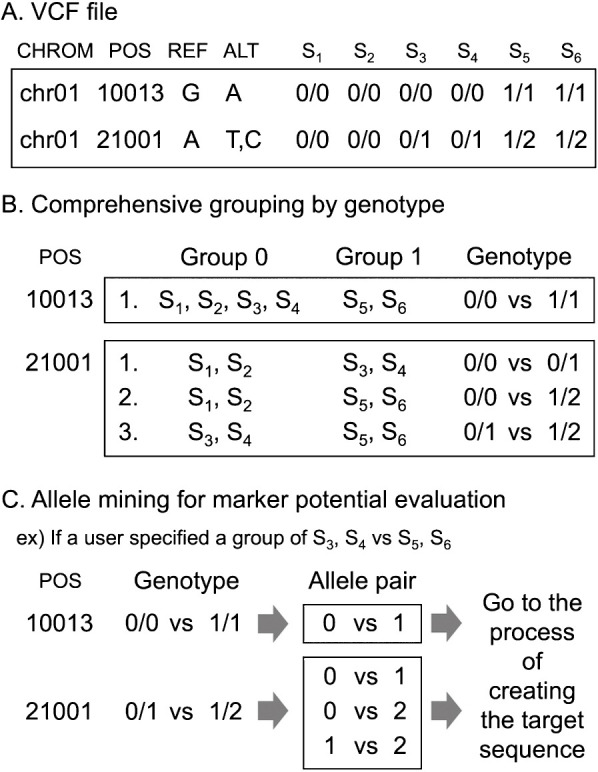
Schematic view of grouping process. (A) An example of VCF genotyping data. (B) V-primer performs a comprehensive grouping on all samples with auto-group mode. (C) V-primer mines potential marker allele pairs according to a user-specified group.

V-primer analyzes multiple samples in a VCF file in two groups (Fig. 2). This grouping allows the researcher to design DNA markers that can be used across multiple samples. Two modes of group processing are implemented: a mode in which the user can specify any two groups and a mode in which the program automatically divides the samples into two groups for exhaustive analysis based on the alleles of the polymorphic site.

V-primer can design primers for three distinct types of DNA markers used in target amplicon sequencing: InDel, CAPS, and SNP markers. The user chooses the marker type and specifies the region of interest for which they wish to design primers.

**Fig. 3.**
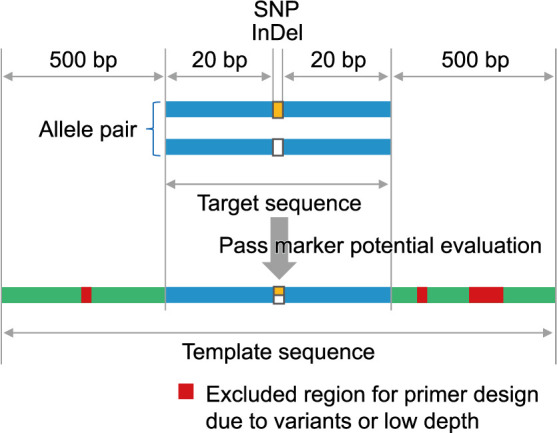
Schematic view of target sequence and template sequence.

To design primers using Primer3 software, a template sequence containing variants that can serve as markers is generated. The target sequences are extracted from the reference sequence to cover each of the 20 bases upstream and downstream of each variant (Fig. 3). The target sequences are then evaluated to determine their utility as markers. For InDel markers, the difference in length of the allele is checked to see if it matches the user-specified InDel size. For CAPS markers, V-primer checks each allele for overlap with sequences recognized by restriction enzymes. Among the two analyzed groups, an allele that a specific enzyme can digest is searched to determine whether it exhibits a polymorphism at the respective locus with both digested and non-digested alleles. After passing this evaluation, a template sequence is generated by extending the target sequence on each side by 500 bases (Fig. 3). In the case of CAPS markers, if there are other restriction enzyme recognition sites in the template sequence with the same recognition site as the target marker site, the sequence upstream or downstream of the recognition site is removed to ensure that there is only one cutting site in the template sequence.

Subsequently, information on regions to be excluded from primer design is added to the template sequence (Fig. 3). Variant information other than the candidate variant within the template sequence is obtained from the VCF file, and regions with polymorphisms among samples or groups are excluded from primer design using Primer3. Additionally, if the option using BAM files is utilized, regions with a read depth below a user-specified value are also excluded from the primer design.

### Primer design step

Then, Primer3 designs primers using the template sequence, targeting regions that do not overlap with the excluded regions (Fig. 3). Users can specify various settings available in Primer3, such as PCR amplicon size and melting temperature (Tm) value.

To guarantee the specificity of the designed primers, V-primer utilizes the BLASTN command of the BLAST+ software to examine the potential for amplification outside the intended target region. The primer pair is removed if any possibility of amplification outside the target region (i.e., the primer pairs are facing each other within 10 kb) is detected. In such cases, the sequence of the removed primer is incorporated into the template sequence as exclusion information, and primer design is repeated. This iterative redesign procedure is repeated until a primer pair specific for the intended target region is detected or until Primer3 can no longer design primers. During the design of primers for SNP markers, V-primer includes a tag sequence specified by the user, CS1 and CS2 in this study, in the 5ʹ end of the primer sequence. The Primer3-py package then evaluates and filters primer pairs showing secondary structures, such as hairpin or primer dimers. If any primer pair fails this test, V-primer redesigns the primers using the abovementioned procedure, ensuring that only high-quality primer pairs are used in the subsequent experiments.

At the conclusion of the pipeline, a tab-delimited file is generated, containing information on marker variants, designed primer sequences, PCR product size, primer Tm value, restriction enzyme names (optionally specified by the user), and fragment sizes after restriction enzyme digestion.

### Validation of designed InDel and CAPS markers

We designed primers in rice (*Oryza sativa*) and foxtail millet (*Setaria italica*) to evaluate the efficacy of primers generated by V-primer. For rice, InDel and CAPS markers were designed to distinguish between the japonica cultivar *Oryza sativa* L. ‘Hitomebore’ and the indica cultivar *Oryza sativa* L. ‘Takanari’ and between ‘Hitomebore’ and the japonica cultivar *Oryza sativa* L. ‘Ginganoshizuku’. For foxtail millet, InDel markers were designed for four millet cultivars. The input VCF files were created using the method described in Supplemental Text 1. To test designed markers, genomic DNA was extracted from the leaves of each variety using 100 mM Tris-HCl buffer containing 10 mM EDTA and 1 M KCl, following the method described by Monna *et al.* (2002). The PCR was performed using GoTaq Green Master Mix (Promega, USA), and restriction enzyme digestion was carried out following the manufacturer’s instructions for the CAPS markers. All PCR products were analyzed for polymorphism by electrophoresis on an agarose gel. Markers that showed the expected pattern of electrophoretic bands were considered available. Further details, including the DDBJ accession number of the sequence reads, are described in Supplemental Text 1.

### SNP selection for multiplex PCR target amplicon sequencing using V-primer

183 SNPs were selected as multiplex PCR targets for amplicon sequencing. First, the short reads of rice cultivars ‘Hitomebore’ and the *japonica* cultivar *Oryza sativa* L. ‘Sasanishiki’ were aligned to the reference genome IRGSP1.0, and a VCF file was generated from the resulting BAM files. Next, this VCF file was used as input to run V-primer in SNP mode with a PCR product size range of 150–200 bp and a minimal depth threshold of >5. For this test case, additional manual filtering criteria were applied based on SNP sites where both parents displayed homozygosity: The GC content of each PCR product was set to 40–60% and PCR product size was 150–175 bp. After applying these filtering criteria, markers were selected at approximately 2-Mb intervals, culminating in the design of 183 pairs of primers. The common tag sequence CS1 (5ʹ-ACACTGACGACATGGTTCTACA-3ʹ) was added to the forward primer, and CS2 (5ʹ-TACGGTAGCAGAGACTTGGTCT-3ʹ) was added to the reverse primer. All primers were ordered from the oPools Oligo Pools service (Integrated DNA Technologies, Inc., USA).

### Library construction and amplicon sequencing

The designed primers were validated using 188 F7 rice recombinant inbred lines (RILs) obtained from a cross between ‘Hitomebore’ and ‘Sasanishiki’. Genomic DNA was extracted from the RILs and the parents using the same method as described above. The first PCR was performed on genomic DNA with the primer pool using Multiplex PCR Assay Kit ver. 2 (Takara Bio, Inc., Japan). The PCR amplicons were then purified, after which Illumina P7/P5 sequences and CS1/CS2 sequences, including custom-designed 8-bp dual indices, were added as primers for a second PCR. The second PCR was performed on 1 ng of purified amplicons using KAPA HiFi HotStart ReadyMix (Kapa Biosystems, Inc., USA). The total 190 barcoded libraries were mixed in equal volumes. The pooled libraries were cleaned up, and size was selected around 300 bp. The pooled library was then sequenced as 2 × 150 paired-end reads on an Illumina MiSeq (Illumina, Inc., USA). Following the method described by Ison *et al.* (2016), we used CS1 (5ʹ-A+CA+CTG+ACGACATGGTTCTACA-3ʹ), CS2 (5ʹ-T+AC+GGT+AGCAGAGACTTGGTCT-3ʹ), and CS2rc (5ʹ-A+GAC+CA+AGTCTCTGCTACCGTA-3ʹ) (locked nucleic acid (LNA) nucleotides are preceded by a + sign) oligonucleotides as custom sequencing primers for Illumina MiSeq. More details are presented in Supplemental Text 1 and Supplemental Fig. 1.

### Genotype calling for multiplex PCR target amplicon sequencing

Raw reads were quality-trimmed using Trimmomatic (Bolger *et al.* 2014). The surviving paired reads were mapped to the IRGSP1.0 rice reference sequence using BWA-MEM (Li and Durbin 2009), and only properly paired alignments were retained using SAMtools (Li *et al.* 2009). Genotype calling on the targeted loci and creation of a VCF file were performed by jointly analyzing 188 RILs and both parents using BCFtools ‘mpileup’ and ‘call’ commands (Li 2011).

## Results and Discussion

In this study, we present a novel software, V-primer, for designing primers for InDel, CAPS, and multiplex SNP markers for target amplicon sequencing. V-primer leverages the commonly used multi-sample variant data summarized in VCF format, enabling high-throughput and efficient primer design. We also demonstrate the reliability and efficacy of V-primer for designing primers for various marker types. First, using rice as an example, we calculated the primer design success rate of our software, which is the number of primers designed for the target conditions within the total number of polymorphisms at the whole genome level, for InDel and CAPS markers, respectively, resulting in 88.8% and 71.6%, respectively (Supplemental Table 1). Next, we used rice and foxtail millet varieties to validate the utility of our designed InDel and CAPS markers. Of the markers designed to distinguish between the *japonica* rice cultivar ‘Hitomebore’ and the *indica* rice cultivar ‘Takanari’, 105 out of 135 InDel markers and 18 out of 26 CAPS markers presented the expected polymorphism pattern by gel electrophoresis (Table 1). Additionally, of 13 InDel markers and 11 CAPS markers designed to distinguish between ‘Hitomebore’ and ‘Ginganoshizuku’, 9 InDel markers and 9 CAPS markers were usable for variety identification (Table 1). Moreover, 39 out of 49 InDel markers designed to differentiate between four foxtail millet varieties displayed the expected polymorphism (Table 1). In both crops, approximately 70–80% of the candidate markers were found to be useful, similar to the range (72.6–86.8%) reported in a previous study of the mInDel pipeline in maize (Lv *et al.* 2016). On the other hand, certain primers failed to amplify products, displayed multiple bands, or failed to demonstrate the intended polymorphism. As V-primer relies on a user-generated VCF file as input, it is conceivable that the markers may not perform as anticipated due to the presence of DNA polymorphisms that are not included in the VCF file. Consequently, we hypothesize that improving the accuracy of the input VCF file may lead to more efficient and effective primer design.

**Fig. 4.**
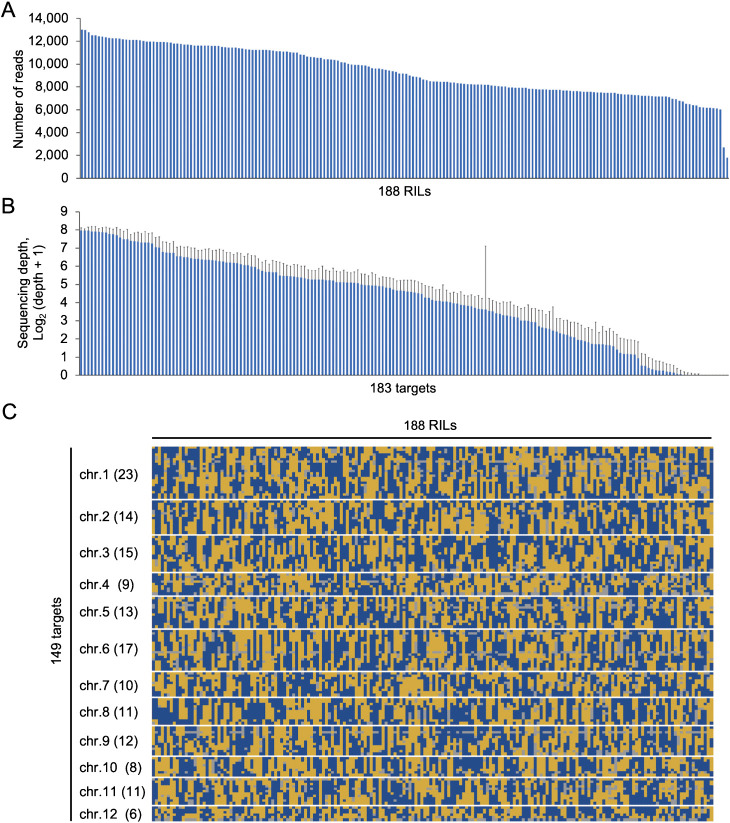
Summary of validation of multiplex PCR target amplicon sequencing using 188 RILs. (A) The number of reads per RIL. The RILs were sorted by number of reads. (B) The average sequencing depth (Log_2_ + 1) per target among 188 RILs. Error bars indicate standard deviations. The targets were sorted by depth. (C) Graphical representation of 188 RILs genotype. Yellow, blue, and gray indicate Hitomebore homozygote allele, Sasanishiki homozygote allele, and missing genotype, respectively. Numbers in parentheses indicate the number of markers for each chromosome.

We evaluated the suitability of 183 SNP markers designed using V-primer for amplicon sequencing in 188 rice RILs derived from a cross between ‘Hitomebore’ and ‘Sasanishiki’. We obtained sufficient read depth per RIL, although two RILs showed slightly lower read counts (Fig. 4a). However, the mean depth per marker varied among markers, with a maximum mean depth of 251 (Log_2_(251+1) = 7.97) (Fig. 4b). Notably, eight markers failed to generate reads in all RILs, and 18 markers had mean depths of 1.5 reads or less, possibly due to differences in PCR efficiency during the multiplex PCR step. In addition, an anomaly was observed for another eight markers, where the genotype of one parent did not appear. Nevertheless, we were able to genotype all 188 RILs using 149 of our designed markers (Fig. 4c), which is sufficient for constructing genetic map (Supplemental Table 2, Supplemental Fig. 2). We performed genomic DNA extraction using a low-cost method (Monna *et al.* 2002) and prepared libraries using low-cost multiplex PCR primers synthesized by oPools Oligo Pools service, enabling relatively inexpensive genotyping of our RILs. Our results suggest that V-primer is a useful tool for developing SNP markers for target amplicon sequencing for genetic research, not only genome wide, but also when designing many SNP markers for specific chromosomes or genomic regions of interest.

Overall, V-primer provides a useful tool for genetics, genomics, and breeding applications, facilitating the efficient and accurate design of InDel, CAPS, and amplicon sequencing marker primers in a single program. V-primer software is available at https://github.com/ncod3/ vprimer and is free to use.

## Author Contribution Statement

S.N. and A.A. designed the research; S.N. wrote the script of V-primer; K.O., C.N., K.I., and H.U. performed the experiments; M.S. and A.A. validated the software; S.N. and A.A. wrote the manuscript; S.N., R.T., and A.A. edited the manuscript.

## Supplementary Material

Supplemental Figures

Supplemental Tables

Supplemental Text

## Figures and Tables

**Table 1.** Summary of validation of InDel and CAPS markers

Crop	Subject	Marker type	Number of markers (% in total)	
			Successful markers	Unsuccessful markers*^a^*
Rice	Hitomebore vs Takanari	InDel	105 (77.8)	30 (22.2)
		CAPS	18 (69.2)	8 (30.8)
Rice	Hitomebore vs Ginganoshizuku	InDel	9 (69.2)	4 (30.8)
		CAPS	9 (81.8)	2 (18.2)
Foxtail millet	Four varieties	InDel	39 (79.6)	10 (20.4)
	Total	InDel	153 (77.7)	44 (22.3)
		CAPS	27 (73.0)	10 (27.0)

*^a^* Unsuccessful markers include those that did not amplify in one or more varieties, had no polymorphisms, and had electrophoresis images with a multi-band pattern.

## References

[B1] Baird, N.A., P.D. Etter, T.S. Atwood, M.C. Currey, A.L. Shiver, Z.A. Lewis, E.U. Selker, W.A. Cresko and E.A. Johnson (2008) Rapid SNP discovery and genetic mapping using sequenced RAD markers. PLoS One 3: e3376. 18852878 10.1371/journal.pone.0003376 PMC2557064

[B2] Bolger, A.M., M. Lohse and B. Usadel (2014) Trimmomatic: A flexible trimmer for Illumina sequence data. Bioinformatics 30: 2114–2120. 24695404 10.1093/bioinformatics/btu170 PMC4103590

[B3] Camacho, C., G. Coulouris, V. Avagyan, N. Ma, J. Papadopoulos, K. Bealer and T.L. Madden (2009) BLAST+: Architecture and applications. BMC Bioinformatics 10: 421. 20003500 10.1186/1471-2105-10-421 PMC2803857

[B4] Cock, P.J.A., T. Antao, J.T. Chang, B.A. Chapman, C.J. Cox, A. Dalke, I. Friedberg, T. Hamelryck, F. Kauff, B. Wilczynski et al. (2009) Biopython: Freely available Python tools for computational molecular biology and bioinformatics. Bioinformatics 25: 1422–1423. 19304878 10.1093/bioinformatics/btp163 PMC2682512

[B5] Danecek, P., A. Auton, G. Abecasis, C.A. Albers, E. Banks, M.A. DePristo, R.E. Handsaker, G. Lunter, G.T. Marth, S.T. Sherry et al. (2011) The variant call format and VCFtools. Bioinformatics 27: 2156–2158. 21653522 10.1093/bioinformatics/btr330 PMC3137218

[B6] Enoki, H., Y. Takeuchi and K. Suzuki (2018) New genotyping technology, GRAS-Di, using next generation sequencer. *In*: Proceedings of the Plant and Animal Genome Conference XXVI. San Diego, CA.

[B7] Henegariu, O., N.A. Heerema, S.R. Dlouhy, G.H. Vance and P.H. Vogt (1997) Multiplex PCR: critical parameters and step-by-step protocol. BioTechniques 23: 504–511. 9298224 10.2144/97233rr01

[B8] Ison, S.A., S. Delannoy, M. Bugarel, T.G. Nagaraja, D.G. Renter, H.C. den Bakker, K.K. Nightingale, P. Fach and G.H. Loneragan (2016) Targeted amplicon sequencing for single-nucleotide-polymorphism genotyping of attaching and effacing *Escherichia coli* O26: H11 cattle strains via a high-throughput library preparation technique. Appl Environ Microbiol 82: 640–649. 26567298 10.1128/AEM.03182-15 PMC4711113

[B9] Li, H. (2011) A statistical framework for SNP calling, mutation discovery, association mapping and population genetical parameter estimation from sequencing data. Bioinformatics 27: 2987–2993. 21903627 10.1093/bioinformatics/btr509 PMC3198575

[B10] Li, H. and R. Durbin (2009) Fast and accurate short read alignment with Burrows-Wheeler transform. Bioinformatics 25: 1754–1760. 19451168 10.1093/bioinformatics/btp324 PMC2705234

[B11] Li, H., B. Handsaker, A. Wysoker, T. Fennell, J. Ruan, N. Homer, G. Marth, G. Abecasis, R. Durbin and 1000 Genome Project Data Processing Subgroup (2009) The sequence alignment/map format and SAMtools. Bioinformatics 25: 2078–2079. 19505943 10.1093/bioinformatics/btp352 PMC2723002

[B12] Lv, Y., Y. Liu and H. Zhao (2016) mInDel: A high-throughput and efficient pipeline for genome-wide InDel marker development. BMC Genomics 17: 290. 27079510 10.1186/s12864-016-2614-5 PMC4832496

[B13] Monna, L., N. Kitazawa, R. Yoshino, J. Suzuki, H. Masuda, Y. Maehara, M. Tanji, M. Sato, S. Nasu and Y. Minobe (2002) Positional cloning of rice semidwarfing gene, *sd-1*: rice “Green Revolution gene” encodes a mutant enzyme involved in gibberellin synthesis. DNA Res 9: 11–17. 11939564 10.1093/dnares/9.1.11

[B14] Ogiso-Tanaka, E., T. Shimizu, M. Hajika, A. Kaga and M. Ishimoto (2019) Highly multiplexed AmpliSeq technology identifies novel variation of flowering time-related genes in soybean (*Glycine max*). DNA Res 26: 243–260. 31231761 10.1093/dnares/dsz005 PMC6589554

[B15] Peterson, B.K., J.N. Weber, E.H. Kay, H.S. Fisher and H.E. Hoekstra (2012) Double digest RADseq: An inexpensive method for *de novo* SNP discovery and genotyping in model and non-model species. PLoS One 7: e37135. 22675423 10.1371/journal.pone.0037135 PMC3365034

[B16] Takeshima, R., E. Ogiso-Tanaka, Y. Yasui and K. Matsui (2021) Targeted amplicon sequencing + next-generation sequencing–based bulked segregant analysis identified genetic loci associated with preharvest sprouting tolerance in common buckwheat (*Fagopyrum esculentum*). BMC Plant Biol 21: 18. 33407135 10.1186/s12870-020-02790-w PMC7789488

[B17] Untergasser, A., I. Cutcutache, T. Koressaar, J. Ye, B.C. Faircloth, M. Remm and S.G. Rozen (2012) Primer3—new capabilities and interfaces. Nucleic Acids Res 40: e115. 22730293 10.1093/nar/gks596 PMC3424584

[B18] Wesołowski, W., B. Domnicz, J. Augustynowicz and M. Szklarczyk (2021) VCF2CAPS—A high-throughput CAPS marker design from VCF files and its test-use on a genotyping-by-sequencing (GBS) dataset. PLoS Comput Biol 17: e1008980. 34014924 10.1371/journal.pcbi.1008980 PMC8186816

[B19] Yuan, J., J. Yi, M. Zhan, Q. Xie, T.T. Zhen, J. Zhou, Z. Li and Z. Li (2021) The web-based multiplex PCR primer design software Ultiplex and the associated experimental workflow: Up to 100- plex multiplicity. BMC Genomics 22: 835. 34794394 10.1186/s12864-021-08149-1 PMC8600765

