# Supplementary material for: V-primer: software for the efficient design of genome-wide InDel and SNP markers from multi-sample variant call format (VCF) genotyping data
Source: Breed Sci. 2023 Sep 9;73(4):415–20. doi: 10.1270/jsbbs.23018 (PMC10722093; doi:10.1270/jsbbs.23018)
Supplement: Supplementary file 1 — Supplemental Figures [file 73_415_s1.pdf]

doi: 10.1270/jsbbs.23018

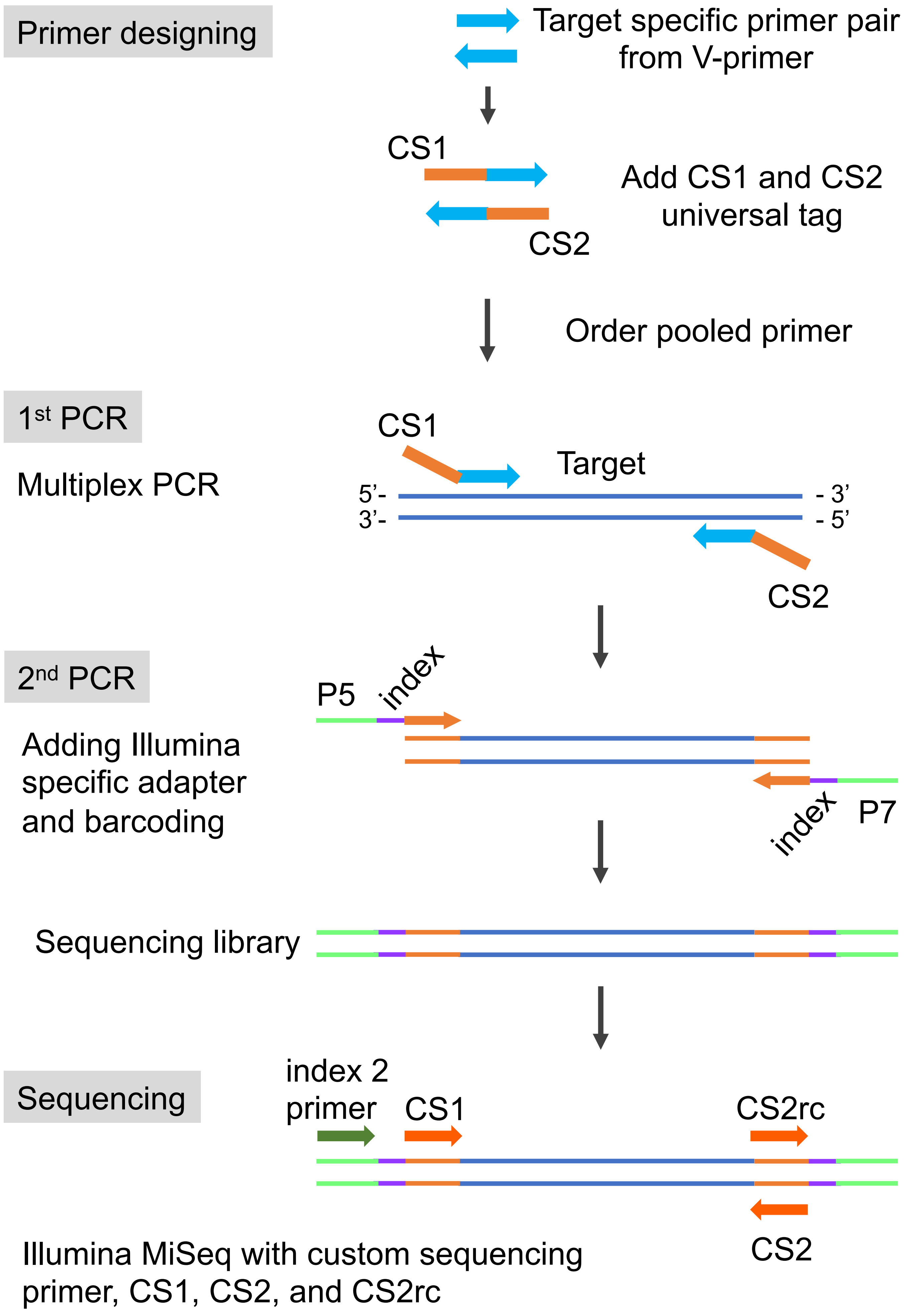

Supplemental Fig. 1. The overview of multiplex PCR target amplicon sequencing in this study.

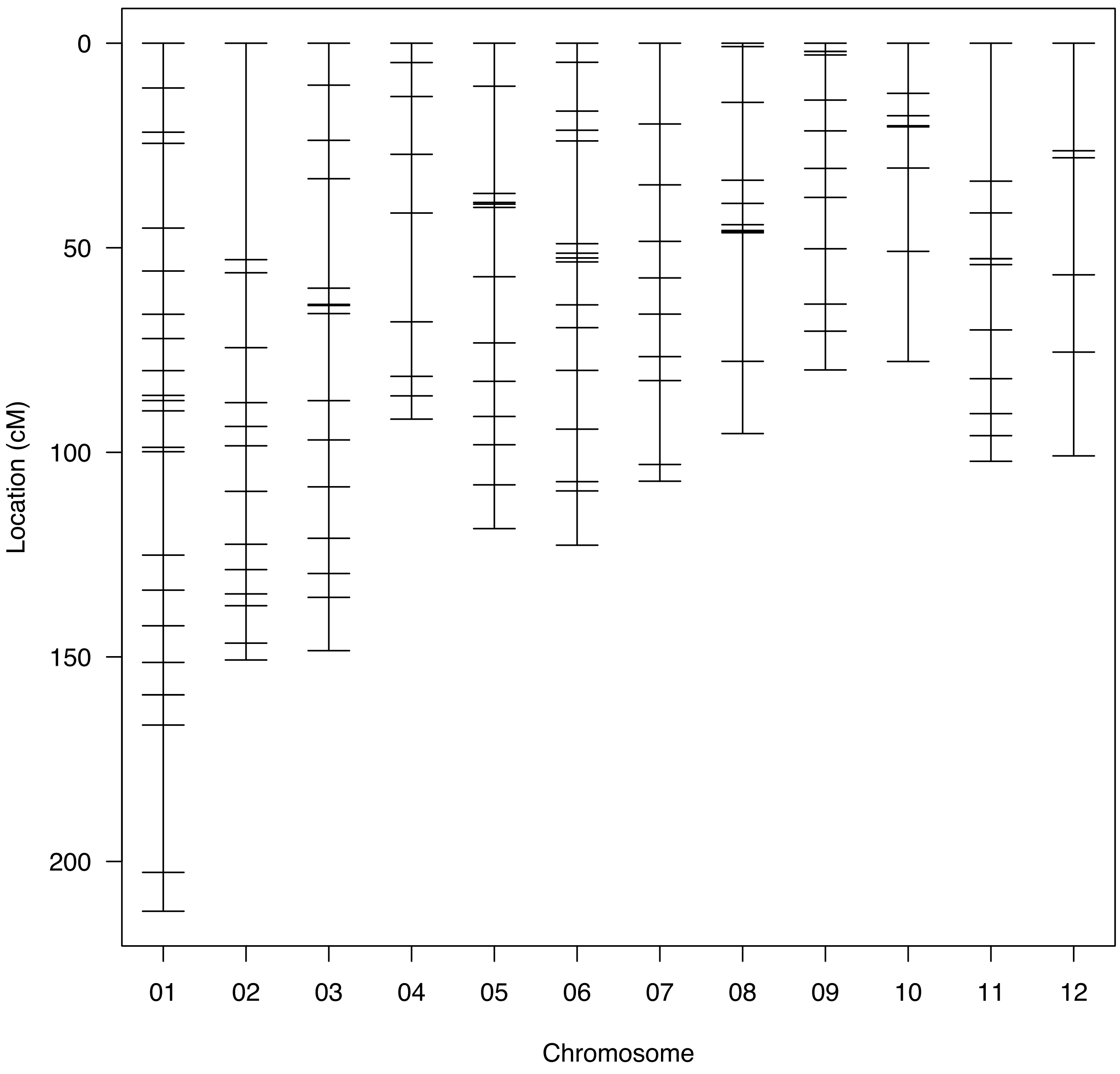

Supplemental Fig. 2. Genetic map of the 149 SNP markers based on rice 12 chromosome using ‘Hitomebore/Sasanishiki’ 188 RILs.

The genetic map was visualized by the R/qtl package (Broman et al. 2003).
