# Supplementary material for: V-primer: software for the efficient design of genome-wide InDel and SNP markers from multi-sample variant call format (VCF) genotyping data
Source: Breed Sci. 2023 Sep 9;73(4):415–20. doi: 10.1270/jsbbs.23018 (PMC10722093; doi:10.1270/jsbbs.23018)
Supplement: Supplementary file 2 — Supplemental Tables [file 73_415_s2.pdf]

doi: 10.1270/jsbbs.23018

Supplemetal Table 1. Primer design efficiency of InDel and CAPS markers using rice varieties Hitomebore and Takanari

Marker type	Number of variants[1)]	Number of filtered variants[2)] (A)	Number of primer designed variants (B)	Primer design efficiency (%) (B/A)
InDel	208,172	7,264	6,450	88.8
CAPS	753,512	225,937	161,796	71.6

1) Variants between Hitomebole and Takanari, excluding variants determined to be heterozygous in either; only SNPs were included in CAPS.

2) The filtering criteria were: InDel markers required a size range of 30 to 100 base pairs; CAPS markers required a restriction enzyme recognition site on the variant.

Supplemental Table 2. Summary of the amplicon sequencing markers.

Marker	Chr.	Physical position (bp)	Genetic distance (cM)
chr01_95019	1	95019	0
chr01_1743229	1	1743229	10.94718662
chr01_3672311	1	3672311	21.75612398
chr01_4276533	1	4276533	24.4748773
chr01_6963610	1	6963610	45.18962697
chr01_8496244	1	8496244	55.68345651
chr01_10479895	1	10479895	66.25053875
chr01_11315694	1	11315694	72.19582532
chr01_12999615	1	12999615	80.03404255
chr01_14953378	1	14953378	86.08116906
chr01_16761727	1	16761727	87.3469291
chr01_18633926	1	18633926	89.86078085
chr01_20590992	1	20590992	98.77322205
chr01_21055909	1	21055909	99.85169993
chr01_24453858	1	24453858	125.121902
chr01_26245475	1	26245475	133.6859439
chr01_27511149	1	27511149	142.3957155
chr01_29043493	1	29043493	151.3524843
chr01_31035257	1	31035257	159.2770265
chr01_31138266	1	31138266	159.2770265
chr01_33604000	1	33604000	166.6572737
chr01_40975340	1	40975340	202.6730391
chr01_42919356	1	42919356	212.1665207
chr02_935258	2	935258	0
chr02_8908716	2	8908716	52.90791753
chr02_9943874	2	9943874	56.11236823
chr02_18968123	2	18968123	74.42245163
chr02_20866259	2	20866259	87.85245398
chr02_22583079	2	22583079	93.67390819
chr02_24580683	2	24580683	98.40808462
chr02_26397040	2	26397040	109.5355995
chr02_28272190	2	28272190	122.4696111
chr02_29561463	2	29561463	128.6699619
chr02_31533726	2	31533726	134.6058721
chr02_32433125	2	32433125	137.4918478
chr02_34416765	2	34416765	146.6351273
chr02_35685408	2	35685408	150.7543037

Supplemental Table 2. continued

Marker	Chr.	Physical position (bp)	Genetic distance (cM)
chr03_5020398	3	5020398	0
chr03_7012241	3	7012241	10.25412276
chr03_10915328	3	10915328	23.73838104
chr03_12774101	3	12774101	33.11948151
chr03_16624895	3	16624895	59.87147603
chr03_18256674	3	18256674	63.83875333
chr03_20080249	3	20080249	64.11183039
chr03_21315824	3	21315824	66.08500038
chr03_24955159	3	24955159	87.36841369
chr03_26292136	3	26292136	96.97345048
chr03_27752991	3	27752991	108.4361727
chr03_30374587	3	30374587	121.0058837
chr03_32435017	3	32435017	129.6165245
chr03_33679081	3	33679081	135.4484532
chr03_36054406	3	36054406	148.4774323
chr04_25887	4	25887	0
chr04_1568950	4	1568950	4.728980753
chr04_3568699	4	3568699	13.03266877
chr04_12924388	4	12924388	27.15974684
chr04_16263067	4	16263067	41.50776876
chr04_20852521	4	20852521	68.10176543
chr04_24113776	4	24113776	81.42915689
chr04_25947245	4	25947245	86.20007399
chr04_27419415	4	27419415	91.88370713
chr05_1583120	5	1583120	0
chr05_4774933	5	4774933	10.51133871
chr05_8640202	5	8640202	36.71921643
chr05_9398906	5	9398906	38.92460716
chr05_13238215	5	13238215	39.33469908
chr05_14526710	5	14526710	40.13921592
chr05_18185382	5	18185382	57.08361397
chr05_20182442	5	20182442	73.25648359
chr05_21932906	5	21932906	82.64752633
chr05_23902344	5	23902344	91.23000415
chr05_25404725	5	25404725	98.14268099
chr05_27369364	5	27369364	107.9432414
chr05_29010920	5	29010920	118.6462477

Supplemental Table 2. continued

Marker	Chr.	Physical position (bp)	Genetic distance (cM)
chr06_2447560	6	2447560	0
chr06_3811724	6	3811724	4.66753861
chr06_5578685	6	5578685	16.59799565
chr06_6520700	6	6520700	21.27568838
chr06_6911532	6	6911532	23.88787325
chr06_12835716	6	12835716	49.00006521
chr06_13625449	6	13625449	51.32587949
chr06_15109915	6	15109915	52.48326089
chr06_16256960	6	16256960	52.48326094
chr06_17405823	6	17405823	53.45893531
chr06_19793253	6	19793253	63.95343091
chr06_20976276	6	20976276	69.52870132
chr06_22631347	6	22631347	79.97362501
chr06_25464801	6	25464801	94.33356573
chr06_27389348	6	27389348	107.1663632
chr06_27696295	6	27696295	109.4316894
chr06_29671063	6	29671063	122.6982137
chr07_3247685	7	3247685	0
chr07_7357762	7	7357762	19.74312095
chr07_15765698	7	15765698	34.62674693
chr07_17928184	7	17928184	48.43157634
chr07_19825453	7	19825453	57.37357815
chr07_21302401	7	21302401	66.21574894
chr07_23242748	7	23242748	76.61265149
chr07_24830619	7	24830619	82.4634549
chr07_28706788	7	28706788	102.9728875
chr07_29683084	7	29683084	107.0568716
chr08_1252296	8	1252296	0
chr08_1659189	8	1659189	0.823772231
chr08_3412934	8	3412934	14.46505879
chr08_5405409	8	5405409	33.49416359
chr08_7393197	8	7393197	39.15506842
chr08_9393044	8	9393044	44.35895634
chr08_11342002	8	11342002	45.77368505
chr08_12891777	8	12891777	46.05363099
chr08_14887346	8	14887346	46.33064849
chr08_21535548	8	21535548	77.76024713
chr08_24525659	8	24525659	95.41790934

Supplemental Table 2. continued

Marker	Chr.	Physical position (bp)	Genetic distance (cM)
chr09_44289	9	44289	0
chr09_1995856	9	1995856	2.019265234
chr09_3977928	9	3977928	2.019265284
chr09_4990944	9	4990944	2.87822213
chr09_7156396	9	7156396	13.89994051
chr09_9148778	9	9148778	21.4284592
chr09_11143715	9	11143715	30.60470273
chr09_13040301	9	13040301	37.6912787
chr09_15479133	9	15479133	50.24708886
chr09_17462193	9	17462193	63.76877702
chr09_18500532	9	18500532	70.38852756
chr09_20296044	9	20296044	79.85928751
chr10_2024031	10	2024031	0
chr10_4001425	10	4001425	12.2558206
chr10_5755367	10	5755367	17.72620264
chr10_8513922	10	8513922	20.1724937
chr10_9513814	10	9513814	20.45410391
chr10_11512761	10	11512761	30.51121269
chr10_17281248	10	17281248	50.88449275
chr10_21177132	10	21177132	77.79371717
chr11_723445	11	723445	0
chr11_5914964	11	5914964	33.71879395
chr11_7845744	11	7845744	41.48372391
chr11_11491127	11	11491127	52.67212632
chr11_13315329	11	13315329	52.67212637
chr11_15298215	11	15298215	54.11926786
chr11_18990712	11	18990712	70.08363556
chr11_20829499	11	20829499	82.00138365
chr11_22476636	11	22476636	90.5412575
chr11_24451957	11	24451957	95.92953256
chr11_26435218	11	26435218	102.1904199
chr12_871838	12	871838	0
chr12_5681701	12	5681701	26.2924715
chr12_6615166	12	6615166	28.00014428
chr12_19286508	12	19286508	56.61156307
chr12_22786386	12	22786386	75.50132356
chr12_27259185	12	27259185	100.869046
