# Supplementary material for: V-primer: software for the efficient design of genome-wide InDel and SNP markers from multi-sample variant call format (VCF) genotyping data
Source: Breed Sci. 2023 Sep 9;73(4):415–20. doi: 10.1270/jsbbs.23018 (PMC10722093; doi:10.1270/jsbbs.23018)
Supplement: Supplementary file 3 — Supplemental Text [file 73_415_s3.pdf]

doi: 10.1270/jsbbs.23018

Supplemental Text 1

Supplementally Materials and Methods

Validation of designed InDel and CAPS markers

For rice, InDel and CAPS markers were designed to differentiate between the japonica cultivar 'Hitomebore' and the indica cultivar 'Takanari', as well as between 'Hitomebore' and the japonica cultivar 'Ginganoshizuku'. To accomplish this, short-read sequencing data from 'Hitomebore', 'Takanari', and 'Ginganoshizuku' were aligned to the reference genome IRGSP1.0 (Kawahara *et al.* 2013) using the BWA software's 'bwa mem' command (https://doi.org/10.48550/arXiv.1303.3997) with the options "-a -T 0". Subsequently, coordinate-sorted BAM files were obtained by employing the 'samtools sort' command from SAMtools v. 1.9 (Danecek *et al.* 2021). By filtering the specified bit in the FLAG field during scanning with the 'samtools view' command, BAM files containing correctly oriented and properly paired mapped reads were extracted. To ensure proper mate pairing, the 'samtools fixmate' command was used to fill in mate coordinates within the BAM files. Lastly, BAM files containing only paired mapped reads were acquired through another execution of the 'samtools view' command. SNP genotype calling was performed, yielding a variant call format (VCF) file. The VCF file generation involved the following steps: (i) utilizing BCFtools v1.9 (Danecek *et al.* 2021) with the 'mpileup' command and options "-a DP,AD,SP,ADF,ADR -B -q 10 -Q 13 -C 50"; (ii) applying the 'call' command from BCFtools with options "-vm -f GQ,GP"; and (iii) executing the 'filter' command from BCFtools with the option "-i 'INFO/MQ>=10'" to filter variants based on a minimum mapping quality (MQ) threshold. The VCF file for InDel genotype was generated using the "run" command and option "--germline" of the SvABA program (Wala *et al.* 2018). The SNP genotype VCF and InDel genotype VCF files were merged using the "concatenate" command of the bcftools program. This mergeed file was used as input to run V-primer in InDel mode with a PCR product size range of 200–400 bp and an InDel size of 30–100 bp. V-primer was also run in CAPS mode with a PCR product size ranging from 300 to 600 bp.

For foxtail millet, InDel markers were designed to identify polymorphisms among four varieties, ‘Yuikogane,’ ‘Shinanotsubuhime,’ ‘Taira,’ and ‘Nisatai-zairai.’ For foxtail millet, InDel markers were designed to identify polymorphisms among four varieties, ‘Yuikogane,’ ‘Shinanotsubuhime,’ ‘Taira,’ and ‘Nisatai-zairai.’ First, short reads of the four foxtail millet varieties were aligned to the reference genome GCA_000263155.2 Setaria_italica_v2.0 (Bennetzen *et al*. 2012), and a VCF file was created from the resulting BAM files in the same manner as for rice. This VCF file was then used as input for a V-primer run in InDel mode with a PCR product size range of 150–200 bp and an InDel size of 30–50 bp.

To test these markers, genomic DNA was extracted from the leaves of each variety using 100 mM Tris-HCl buffer containing 10 mM EDTA and 1 M KCl, following the method described by Monna *et al*. (2002). For PCR amplification, a 5-μL reaction mixture was prepared with 0.5 μL of genomic DNA, 0.5 μL of each 10 pM forward and reverse primer, and 2 μL of 2× GoTaq Green Master Mix (Promega, USA). The PCR conditions were as follows: initial denaturation at 94°C for 5 min, 35 cycles of denaturation at 94°C for 30 s, primer annealing at 60°C for 30 s, and extension at 72°C for 30 s, followed by a final incubation at 72°C for 3 min. For the CAPS markers, restriction enzyme digestion was carried out following the manufacturer's instructions. All digested PCR products were analyzed for polymorphism by electrophoresis on a 3% (w/v) agarose gel.

Sequence reads (fastq files) for each sample are available from the following DDBJ accessions.

1) Rice

Hitomebore: DRR391466, DRR391467, DRR391468, DRR391469, DRR391470

Takanari: DRR480536, DRR480537, DRR480538, DRR480539, DRR480540, DRR480541, DRR480542

Ginganoshizuku: DRR480543, DRR480544

2) Foxtail millet

Yuikogane: DRR480546

Shinanotsubuhime: DRR480547

Taira: DRR480548

Nisatai-zairai: DRR315791

Library construction and amplicon sequencing

The designed primers were validated using 188 F_7 rice recombinant inbred lines (RILs) obtained from a cross between Hitomebore and Sasanishiki. Genomic DNA was extracted from the RILs and the parents using the same method as described above, and its concentration was measured using a NanoDrop One spectrophotometer (Thermo Fisher Scientific, Inc., USA). The first PCR was performed on genomic DNA with the primer pool using a reaction mixture containing 10 ng of genomic DNA, 1 µL of the primer pool (0.05 µM each primer), 2.5 µL of 2× Multiplex PCR buffer, and 0.05 µL of Multiplex PCR enzyme mix (Multiplex PCR Assay Kit ver. 2, Takara Bio, Inc., Japan), in a final volume of 5 µL. The first PCR was conducted with the following conditions: initial denaturation at 94°C for 1 min, 30 cycles of denaturation at 94°C for 30 s, and primer annealing and extension at 60°C for 4 min, followed by a final incubation at 72°C for 10 min. The PCR amplicons were then purified using 1.8× volume of Agencourt AMpure XP (Beckman Coulter, Inc., USA), after which Illumina P7/P5 sequences and CS1/CS2 sequences, including custom-designed 8-bp dual indices, were added as primers for a second PCR. The second PCR mixture contained 1 ng of purified DNA products, 0.75 µL of each 5 µM index primer, and 5 µL of 2× KAPA HiFi HotStart ReadyMix (Kapa Biosystems, Inc., USA), in a final volume of 10 µL. The second PCR was carried out under the following conditions: initial denaturation at 95°C for 5 min, 16 cycles of denaturation at 98°C for 20 s, primer annealing at 65°C for 15 s, and extension at 72°C for 1 min, followed by a final incubation at 72°C for 5 min. The total 190 barcoded libraries were mixed in equal volumes. The pooled libraries were cleaned up with Agencourt AMpure XP (Beckman Coulter, Inc., USA) beads following the manufacturer's instructions, and size was selected around 300 bp. After size selection, the multiplexed library was eluted in 10 mM Tris-HCl (pH 8.0). The final concentration of the multiplexed library was determined by quantitative PCR using a Library Quantification Kit (Takara Bio USA, Inc., USA) and diluted to 8 pM. The multiplexed library, consisting of 190 samples, was then sequenced as 2× 150 paired-end reads on an Illumina MiSeq instrument using a MiSeq Reagent Micro Kit v2 (300 cycles, Illumina,

Inc., USA). Following the method described in Ison *et al*. (2016), we used CS1, CS2, and CS2rc oligonucleotides for custom sequencing primers, which were synthesized with locked nucleic acid nucleotides.

An overview of the target amplicon sequencing in this study is presented in Supplemental Figure 1.

Literature Cited

Bennetzen, J.L., J. Schmutz, H. Wang, R. Percifield, J. Hawkins, A.C. Pontaroli, M. Estep, L. Feng, J.N. Vaughn, J. Grimwood *et al*. (2012) Reference genome sequence of the model plant Setaria. Nat Biotechnol 30: 555–561.

Danecek, P., J.K. Bonfield, J. Liddle, J. Marshall, V. Ohan, M.O. Pollard, A. Whitwham, T. Keane, S.A. McCarthy, R.M. Davies *et al*. (2021) Twelve years of SAMtools and BCFtools. GigaScience. 10: giab008.

Ison, S.A., S. Delannoy, M. Bugarel, T.G. Nagaraja, D.G. Renter, H.C. den Bakker, K.K. Nightingale, P. Fach and G.H. Loneragan (2016) Targeted amplicon sequencing for single-nucleotide-polymorphism genotyping of attaching and effacing *Escherichia coli* O26: H11 cattle strains via a high-throughput library preparation technique. Appl Environ Microbiol 82: 640–649.

Kawahara, Y., M. de la Bastide, J.P. Hamilton, H. Kanamori, W.R. McCombie, S. Ouyang, D.C. Schwartz, T. Tanaka, J. Wu, S. Zhou *et al*. (2013) Improvement of the *Oryza sativa* Nipponbare reference genome using next generation sequence and optical map data. Rice (N Y) 6: 4.

Monna, L., N. Kitazawa, R. Yoshino, J. Suzuki, H. Masuda, Y. Maehara, M. Tanji, M. Sato, S. Nasu and Y. Minobe (2002) Positional Cloning of Rice Semidwarfing Gene, *sd-1*: Rice "Green Revolution Gene" Encodes a Mutant Enzyme Involved in Gibberellin Synthesis. DNA Res 9: 11–17.

Wala, J.A., P. Bandopadhayay, N.F. Greenwald, R. O'Rourke, T. Sharpe, C. Stewart, S. Schumacher, Y. Li, J. Weischenfeldt, X. Yao *et al*. (2018) SvABA: Genome-wide detection of structural variants and indels by local assembly. Genome Res 28: 581–591.
